# A Velocity Measurement Method Based on Charge Induction

**DOI:** 10.3390/s23031238

**Published:** 2023-01-21

**Authors:** Yangbin Chi, Ziyu Fan, Shufan Wang, Limin Zhang

**Affiliations:** School of Electronic Science and Engineering, Nanjing University, Nanjing 210023, China

**Keywords:** charge induction, velocity measurement, average velocity, passive detection

## Abstract

In this paper, based on the principle of charge induction, a new velocity measurement method is proposed. A moving target generates a low-frequency electric field, which can be induced with an electrode and detection frontend. Velocity measurements are achieved by placing two electrodes at a fixed distance to detect the characteristic times. Firstly, the electric field generated by the moving target is modeled, and the theoretical output of the detection frontend is obtained via a simulation of the target passing by a single electrode. Then, according to the theoretical output, the velocity measurement simulation results of double electrodes are given for various driving conditions, such as a single vehicle driving in a single lane, a single vehicle changing lanes, two vehicles driving close together, and a multiple-vehicle situation. Finally, the above driving conditions are experimentally verified in sunny weather, windy and rainy weather, and a night environment.

## 1. Introduction

For moving targets, the current traditional velocity measurement technologies mainly include microwave radar technology [1,2,3], laser technology [4,5,6], ground-sensing technology [7,8,9], and video camera technology [10,11,12,13,14]. Microwave radar technology is mainly based on the Doppler effect, which is vulnerable to radio waves and has a low accuracy [15]. Laser technology calculates velocity through the distance and time intervals of two measurements. Since the laser beam must be perpendicular to the reflective surface, this technology has disadvantages, such as a poor resistance to occlusion, a high equipment cost, and the inability to achieve lane-change velocity measurements [16]. Ground-sensing technology uses the time interval required for the vehicle to pass two fixed inductive loop detectors in order to calculate velocity. However, it has high installation and maintenance costs, as it needs to be installed under the road. In addition, it also has disadvantages, such as being easily damaged due to road surface deformation and the inability to achieve lane-change velocity measurements. Video camera technology is the most widely used velocity measurement method, and it uses target tracking or a virtual coil. The target tracking method first selects the target feature points for identification, then uses an algorithm to track the target, and finally calculates the velocity through the displacement between the image frames. The virtual coil method actively places virtual coils in the measurement area and calculates the velocity by detecting a trigger that is activated from when a vehicle passes the coil. Although the video camera technology works relatively well, it is easily affected by environmental factors, such as light and weather. The velocity measurement accuracy depends on the resolution of the camera and the background noise reduction of the processing algorithms [17]. Furthermore, when there are multiple vehicles driving in multiple lanes, it is often difficult to measure velocity [18].

In addition to the traditional velocity measurement technologies, some innovative velocity measurement technologies have also been proposed.

To measure vehicle velocity, a measurement method has been proposed whereby two magnetic sensors are placed with fixed relative positions on the roadside. When the vehicle velocity is in the range of 5 m/s to 27 m/s, the velocity measurement error is less than 2.5%. However, this method requires a complex algorithm to obtain a high signal-to-noise ratio in order to ensure accuracy. Moreover, when using this method, large vehicles lead to large errors because of the complex magnetic fields [19].

Another velocity measurement method combining infrared and ultrasonic sensors has been proposed, and it is relatively simple and low-cost but has poor occlusion resistance. Additionally, complex algorithms are required to overcome noise and environmental effects [20].

A velocity measurement method based on Wi-Fi technology has been proposed, and it uses Wi-Fi signals emitted by roadside wireless devices to measure velocity by analyzing the impact of vehicles passing by the signal, with an error of less than 2.6 km/h. However, due to the interference of many other signals, the accuracy of the velocity measurement is greatly affected by the environment. Vehicle velocity above 60 km/h cannot be measured because of the limited sampling rate of the system [21].

It is difficult to satisfy multiple factors at the same time with the current velocity measurement technologies, such as accuracy, equipment cost, algorithm complexity, and anti-interference ability. Therefore, the aim of the paper is to propose a method that can measure vehicle velocity with a high accuracy, a good stability, a low cost, a simple algorithm, and a strong anti-interference ability in several working situations, such as when a vehicle changes lanes.

When a target moves, it generates a low-frequency electric field, which reflects the position and state information of the target. As early as the last century, a ship’s electric field was an important object in the field of electric field detection.

A ship’s electric field is mainly caused by the body’s corrosion and the electromotive force generated by the hull cutting the geomagnetic field lines, the rotation of the propeller, and the spatial magnetic flux changes due to the magnetic hull motion [22]. At present, the commonly used modeling technique for a ship’s electric field is magnetic modeling (i.e., magnetic dipole model) because of its simple structure [23]. In fact, it is very difficult to completely rely on theoretical calculations to study the electric field characteristics of ships because of various complicated influencing factors. Therefore, some properties are usually obtained through practical experiments [24].

The principle that the bodies of ships and vehicles can usually be regarded as magnetic dipoles is due to the fact that the magnetic anomaly of objects containing iron is much higher than that of the surrounding medium, leading to the distortion of the magnetic flux lines of the Earth’s magnetic field, thereby causing changes in the surrounding magnetic field. However, since different types of vehicles have different metal components and structures, they can be expected to produce different magnetic field signatures. For simplicity, the electric field of a moving vehicle is treated as a single magnetic dipole model [25], which can be induced by a metal electrode and a charge sensor circuit with ultra-high input impedance [26]. Previous studies conducted by our group have shown the feasibility of the recognition of hand motion direction and rotor direction based on charge induction [27,28].

Therefore, a velocity measurement method is proposed based on the charge induction principle, where two metal electrodes are placed in the moving direction of the target and connected to detection frontends, and a velocity measurement is obtained via the time characteristic of the charge change when a target passes by the two electrodes. The significant advantages of this method are that it can work effectively in a passive mode whether it is day or night, it is less affected by light and weather, and it can measure the velocity of vehicles changing lanes; it also has a low cost and is easy to implement.

This paper is organized as follows: Section 2 introduces the theoretical derivation of induced charge on the surface of a metal electrode caused by a moving vehicle. In Section 3, a two-electrode velocity measurement method is proposed based on charge induction, where the feasibility of several scenarios is analyzed in detail. In Section 4, corresponding experiments are implemented to verify the effectiveness of the proposed method. Finally, conclusions are drawn in Section 5.

## 2. Moving Target Model

A moving vehicle can be modeled as a moving magnet. A moving magnet creates an induced electric field in the surrounding space. The motion of the target can be analyzed by sensing the changing charge caused by the low-frequency electric field. A magnetic dipole is the most basic simulation unit for magnetism model analyses. For convenience, a single magnetic dipole moving in the direction of the magnetic moment is used to establish the vehicle body motion model.

**Figure 1:**
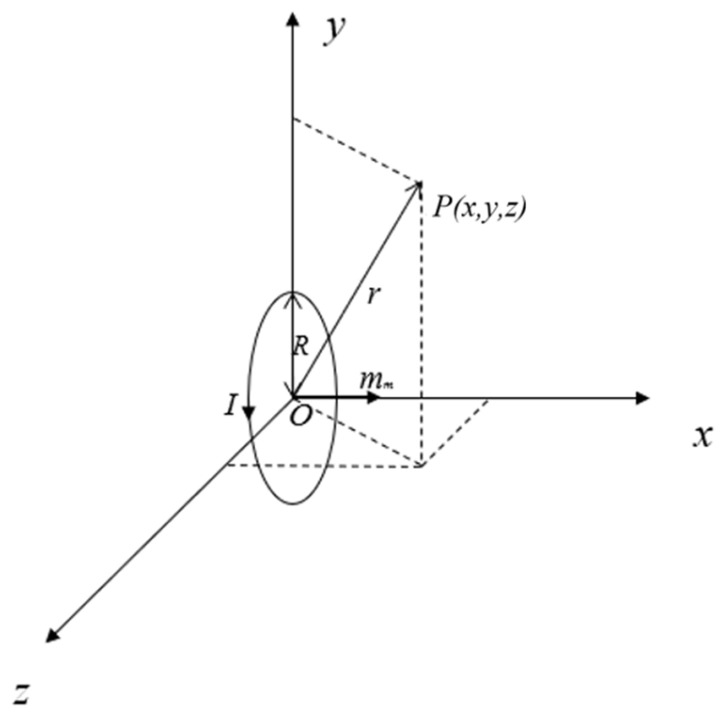
A magnetic dipole with the magnetic moment along the x-direction.

A magnetic dipole refers to a planar current-carrying loop of any shape with a small area. As shown in Figure 1, for a magnetic dipole whose magnetic moment is along the x-direction, the current path is assumed to be a circle with radius *R*, and it is assumed that the loop radius *R* of the source current I is much smaller than the distance r from the point to be measured *P* to the center of the source current loop. Then, the magnetic field *B* generated by the magnetic dipole at point *P(x, y, z)* in space can be obtained by integrating the current loop according to the Biot–Savart law as follows:

(1) $$\left\{\begin{array}{c} B_{x}=K\left(\frac{3x^{2}}{r^{5}}-\frac{1}{r^{3}}\right)\\ B_{y}=K\frac{3xy}{r^{5}}\\ B_{z}=K\frac{3xz}{r^{5}} \end{array}\right.,$$

$$B=\sqrt{B_{x}{}^{2}+B_{y}{}^{2}+B_{z}{}^{2}}$$

where *K* is equal to $\frac{\mu_{0}m_{m}}{4\pi }$, *r* is equal to $\sqrt{x^{2}+y^{2}+z^{2}}$, the vacuum permeability $\;\mu_{0}$ $\mathrm{is}\;4\pi \times 10^{-7}\;Tm/A$, and $m_{m}$ is the magnetic moment.

**Figure 2:**
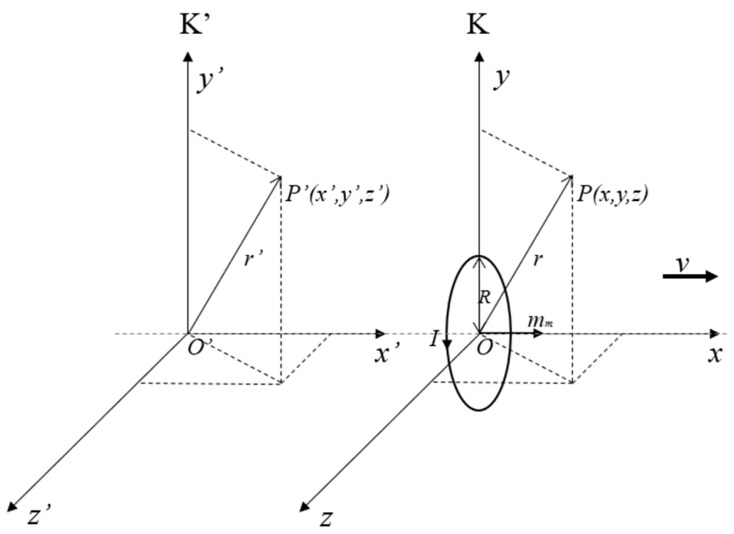
Coordinate model of moving magnetic dipole.

The moving magnetic dipole model is shown in Figure 2. Let K’ and K be two inertial Cartesian coordinate systems; the K’ system is stationary, and the K system moves at a uniform velocity relative to the K’ system along the x’ direction at velocity *v*. The origin of the K system is located at the center of the magnetic dipole, and the magnetic dipole moves along the x direction with the uniform velocity *v* in the K system. At *t = t’* = 0, the origin O of the K system is coincident with that of the K’ system.

In the K system, since the magnetic moment is along the x-axis direction, the magnetic field component at any point *P(x, y, z)* in the K system can be obtained as follows:

(2) $$\left\{\begin{array}{c} {E_{x}}^{\prime }=E_{x}\\ E_{y}^{\prime }=\gamma \left(E_{y}+vB_{z}\right)\\ E_{z}^{\prime }=\gamma \left(E_{z}-vB_{y}\right) \end{array}\right.,$$

where $\gamma =\frac{1}{\sqrt{1-{\frac{v}{c}}^{2}}}\approx 1$, and *c* is the velocity of light in the medium. Since the K system is relatively stationary with the magnetic dipole, leading to $E_{x}=E_{y}=E_{z}=0$, the electric field component of the moving magnetic dipole in the K’ system can be simplified as

(3) $$\left\{\begin{array}{c} E_{x}^{\prime }=0\\ E_{y}^{\prime }=\frac{vK}{r^{5}}\times 3xz\\ E_{z}^{\prime }=\frac{vK}{r^{5}}\times 3xy \end{array}\right.,$$

According to the coordinate transformation relationship, $z^{\prime }=z,y^{\prime }=y,x^{\prime }=x+vt$, the electric field component is obtained as follows:

(4) $$\left\{\begin{array}{c} E_{x}^{\prime }=0\\ E_{y}^{\prime }=\frac{vK}{r^{5}}\times 3\left(x^{\prime }-vt\right)z^{\prime }\\ E_{z}^{\prime }=\frac{vK}{r^{5}}\times 3\left(x^{\prime }-vt\right)y^{\prime } \end{array}\right.,$$

The charge is generated on the surface of the metal electrode due to electrostatic induction, and the relationship between the amount of induced charge and the electric field intensity can be expressed as

(5) $$Q={∯}_{A}^{\;}\varepsilon \vec{E}\cdot d\;\vec{A},$$

where *Q* is the amount of induced charge on the conductor surface, and *A* is the surface area of the metal electrode.

In the car velocity measurement model, it is assumed that the motion direction is along the x′ axis, the detection electrode is placed on the positive half-axis of the z′ axis, and the electrode plane is perpendicular to the z′ direction. Then, the inducted charge on the electrode is mainly from the electric field component *E_z′_*. Since the radius of the detection electrode is much smaller than the detection distance z′, for the convenience of calculation, the electric field detected by the electrode is regarded as a uniform electric field; then, Equation (5) can be simplified as

(6) $$Q=\varepsilon E_{Z}^{\prime }A,$$

**Figure 3:**
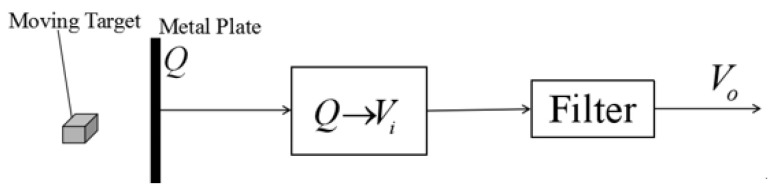
Process of moving target detection.

To detect the amount of induced charge, the metal electrode is connected to the capacitor *C_i_*, and the voltage *V_i_* across the capacitor is proportional to the amount of induced charge *Q*. In order to reduce the influence of noise, *V_i_* is filtered to finally obtain *V_o_*, and the processing process is shown in Figure 3.

According to Equation (4), the output voltage when the target moves horizontally is

(7) $$V_{0}\left(m_{m},x^{\prime },y^{\prime },z^{\prime }\right)=\frac{\varepsilon \mu_{0}m_{m}v\mathrm{HA}}{4\pi C_{i}{\sqrt{{\left(x^{\prime }-vt\right)}^{2}+y^{\prime 2}+z^{\prime 2}}}^{5}}\times 3\left(x^{\prime }-vt\right)y^{\prime },$$

where *H* is the gain of the filter circuit.

To illustrate the above analysis, some simulations are given below.

**Figure 4:**
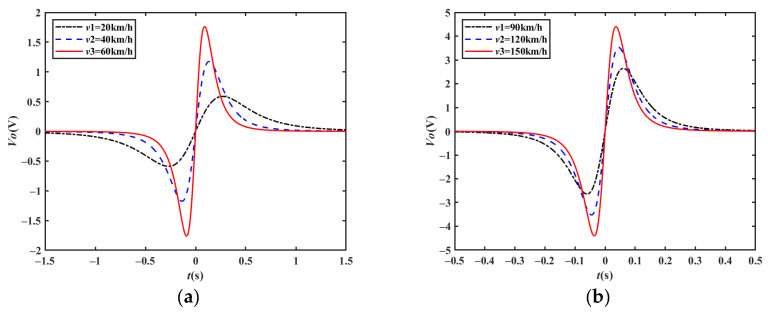
Simulation output waveforms at different velocities: (**a**) velocity at 20 km/h, 40 km/h, and 60 km/h; (**b**) velocity at 90 km/h, 120 km/h, and 150 km/h.

Assuming that the magnetic moment of the vehicle *m_m_* is 1 A/m^2^, *A* = 0.01 m^2^, *C_i_* = 10 pF, *H* = 1, the position of the detection electrode is *x′* = 0, the distance between the electrode and the driving lane is *z′* = 3 m, and the height of the electrode relative to the center of the magnetic moment is *y′* = 0.1 m; the simulation output waveforms from 20 km/h to 150 km/h are shown in Figure 4, where the output voltage *V_o_* is a center-symmetric double-peak waveform, and the time point corresponding to the center-symmetric point is when the moving target passes through the center of the detection electrode. When the electrode detection distance remains unchanged and the target velocity increases, the width of the output waveform becomes narrower, and the peak amplitude is proportional to the vehicle velocity.

**Figure 5:**
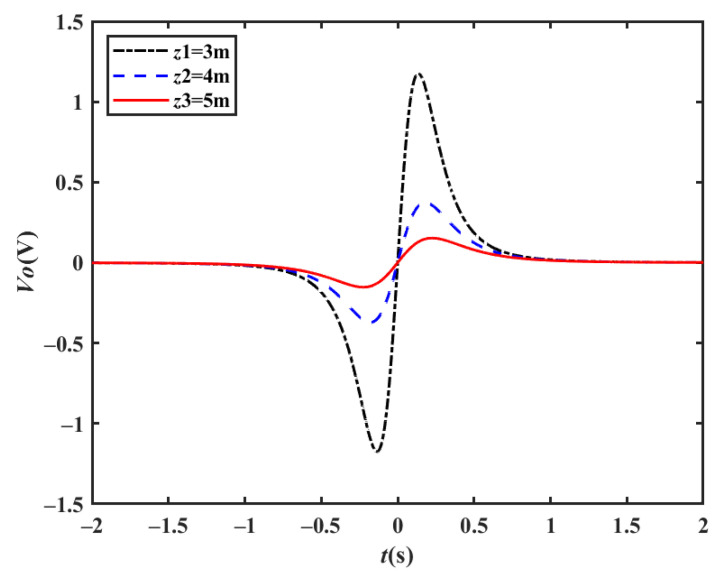
Simulation output waveforms at different detection distances (*v* = 40 km/h).

When the distance *z′* between the detection electrode and the driving direction changes, the output waveform for the moving target velocity *v* = 40 km/h is as shown in Figure 5, where the waveform amplitude decreases, and the waveform width does not change much, with the peak coordinates slightly delayed.

**Figure 6:**
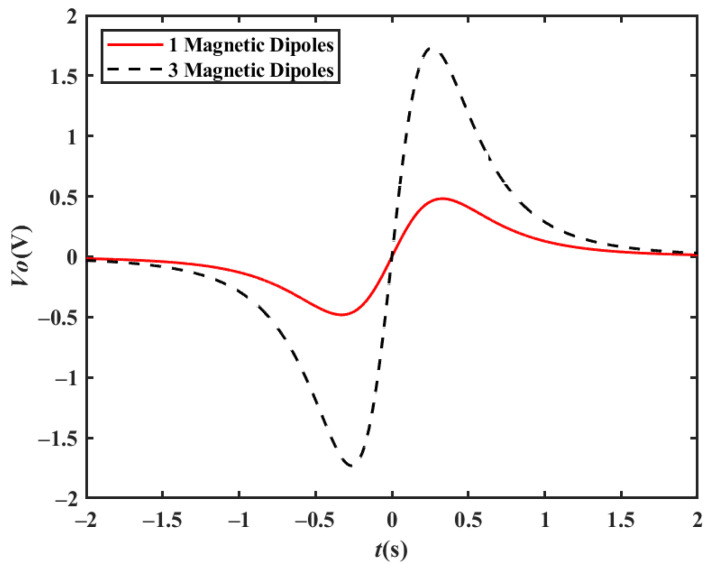
Simulation output waveforms of multi-magnetic dipole model(*v* = 40 km/h).

Due to the diversity of vehicle body types, multiple magnetic dipole models are required for vehicle bodies with a large size and a complex structure. Therefore, three magnetic dipoles distributed along the x-axis with a distance of 1 m are examined in a simulation. Assuming that the velocity of the vehicle is 20 km/h, the position of the detection electrode is *x′* = 0, the distance between the electrode and the driving lane is *z′* = 3 m, and the height of the electrode relative to the magnetic moment center is *y′* = 0.1 m; the output waveforms for a single magnetic dipole and three magnetic dipoles are shown in Figure 6. It can be seen from the simulation results that multiple magnetic dipole models only affect the waveform size and that the waveform characteristics remain unchanged.

**Figure 7:**
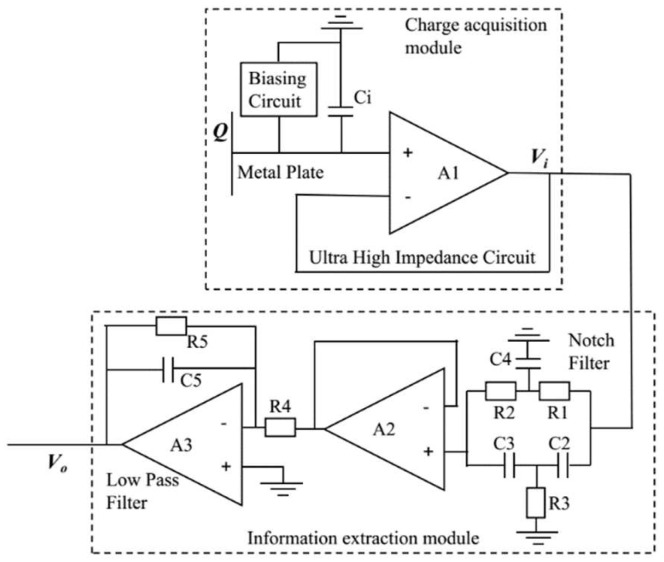
Circuit diagram of detection frontend.

Furthermore, the experimental verification of the magnetic dipole model is implemented, and the detection frontend is shown in Figure 7. The high-impedance circuit [26] proposed by our group is used to convert the induced charge *Q* on the metal plate into voltage *V_i_*; the input capacitance *C_i_* is about 10 pF, and the input resistance is about 100 GΩ, where the input capacitance *C_i_* is the equivalent input capacitance, including the parasitic capacitance circuit and the frontend operation amplifier input capacitance. A notch filter of 50 Hz and a low-pass filter with a characteristic frequency of about 50 Hz are used to suppress noise, amplify the signal, and output signal *V_o_*.

**Figure 8:**
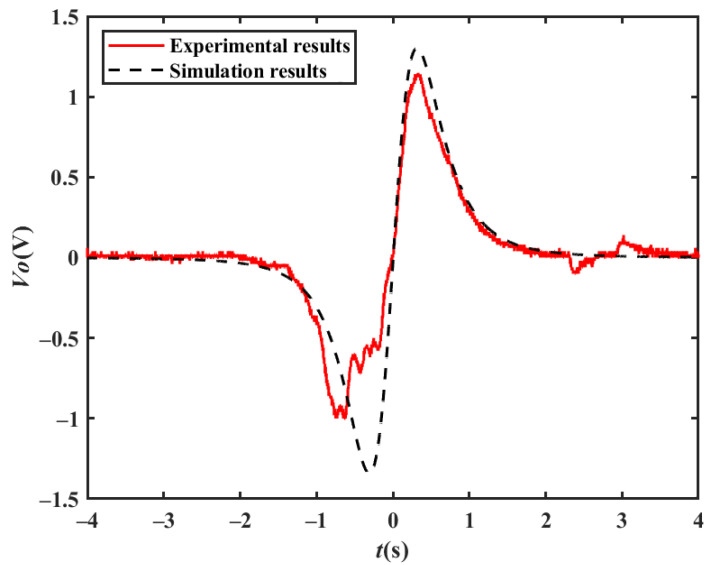
Comparison of experimental and simulation results.

In the experiment, the area of the electrode is about 0.005 m^2^, the distance between the electrode and the driving lane is about 2.75 m, the electrode height is 1 m from the ground, and the filter circuit gain is 10. In the theoretical simulation, the magnetic moment of the vehicle is *m_m_* = 1 A/m^2^, the velocity *v* = 16 km/h, the electrode area *A* = 0.005 m^2^, the capacitance *Ci* = 10 pF, the filter circuit gain *H* = 10, the detection electrode position *x′* = 0, the distance between the electrode and the driving lane is *z′* = 2.75 m, and the height of the electrode relative to the center of the magnetic moment is *y′* = 0.05 m. Figure 8 shows the experimental results and the simulation results, where the characteristics of the experimental results are basically consistent with those of the theoretical results, but the peak value is different, which may be due to the inaccurate setup of the size and specific location of the magnetic moment of the car.

## 3. Velocity Measurement Method

### 3.1. Mechanism

**Figure 9:**
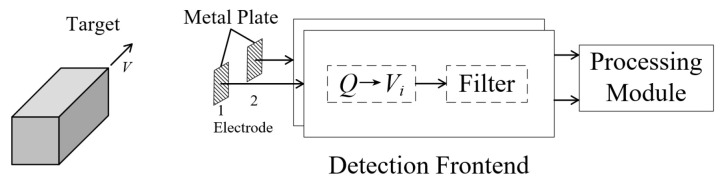
Schematic diagram of the velocity measurement model.

**Figure 10:**
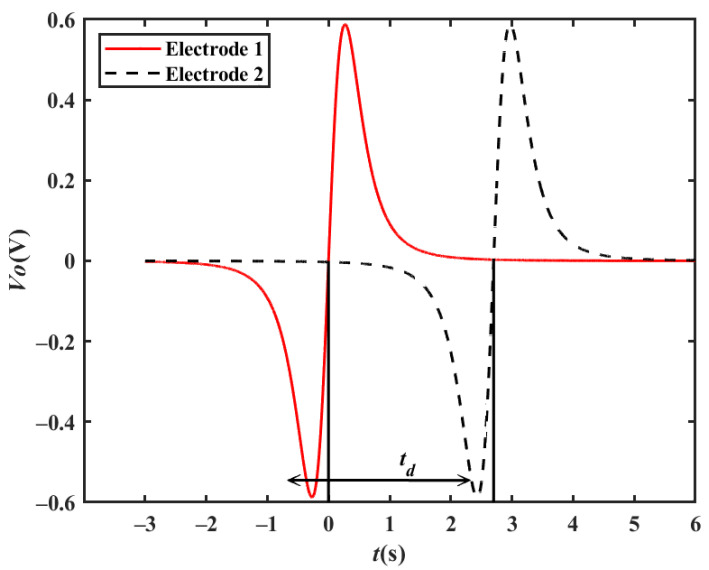
Velocity measurement waveforms.

Based on the output waveform of one electrode, two metal electrodes are used to simultaneously sense the charge change when a target passes by in order to obtain a velocity measurement. As shown in Figure 9, two metal electrodes with a certain distance are placed in the moving direction of the target, and they are connected to two detection frontends. The two metal electrodes sense the charge change when the target passes by, and then the two detection frontends convert the induced charges into two output waveforms, which have a time delay between them. Finally, the processing module calculates the velocity according to the time delay *t_d_* between the two output waveforms, as shown in Figure 10. By setting the distance between the two polar plates as *d*, the moving velocity *v* of the target can be calculated using Equation (8), where the vehicle passes electrodes 1 and 2 successively.


(8) $$v=\frac{d}{t_{d}}\;,$$

There are many methods for solving the time delay *t_d_*. The cross-correlation algorithm is used in this paper. Assuming that the output waveforms of the two electrodes are *V_o1_(n)* and *V_o2_(n)* and that the length is *N*, the cross-correlation of the two signals R_V_o1_V_o2__ is


(9) $$R_{V_{o1}V_{o2}}\left(n\right)=\frac{1}{N}\sum_{m=0}^{N-1}V_{o1}\left(m\right)V_{o2}\left(m-n\right),$$

From a physical perspective, the cross-correlation operation R_V_o1_V_o2__ reflects the similarity between the two signals and the relative translation of the signal *n*. In velocity measurement applications, this algorithm can be used to obtain the corresponding translation amount *n* when the waveforms overlap, and then this can be divided by the sampling rate *f_s_* to obtain the time delay difference *t_d_*. Combining Equations (8) and (9), the velocity *v* can be obtained as follows:


(10) $$v=\frac{d\cdot fs}{n}\;,$$

**Figure 11:**
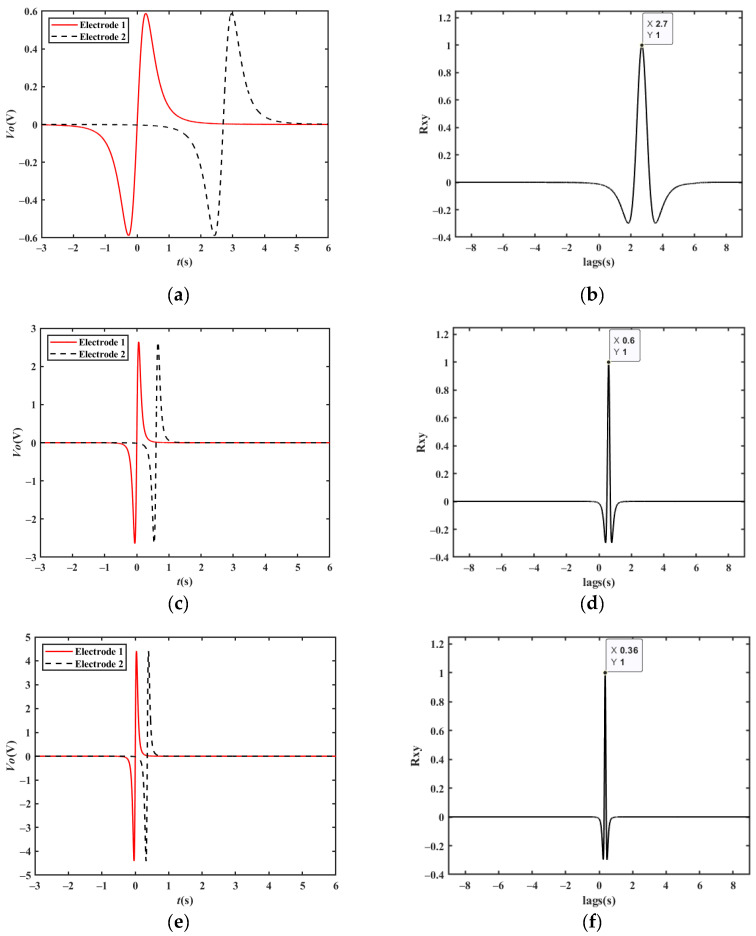
Simulation results of a single vehicle passing by two electrodes: (**a**) simulation results at a velocity of 20 km/h; (**b**) cross-correlation results at a velocity of 20 km/h; (**c**) simulation results at a velocity of 90 km/h; (**d**) cross-correlation results at a velocity of 90 km/h; (**e**) simulation results at a velocity of 150 km/h; (**f**) cross-correlation results at a velocity of 150 km/h.

Figure 11a shows the simulated output waveform of two electrodes with a distance of 15 m when the vehicle velocity is 20 km/h. The cross-correlation result is shown in Figure 11b. The *t_d_* corresponding to the peak point is approximately 2.700 s. Therefore, the vehicle velocity can be calculated to be 20.00 km/h, which is consistent with the set value.

When the vehicle travels in the reverse direction at a velocity of −20 km/h, the *t_d_* corresponding to the peak point is approximately −2.700 s, and the vehicle velocity can be calculated to be −20.00 km/h, which is consistent with the set value. Similarly, the simulation results of 90 km/h and 150 km/h shown in Figure 11c–f are also in line with expectations.

Based on the above analysis, the velocity measurement method in this paper can realize the velocity measurement of two-way driving vehicles.

### 3.2. Simulation of Different Situations

#### 3.2.1. Single Vehicle Changing Lanes

**Figure 12:**
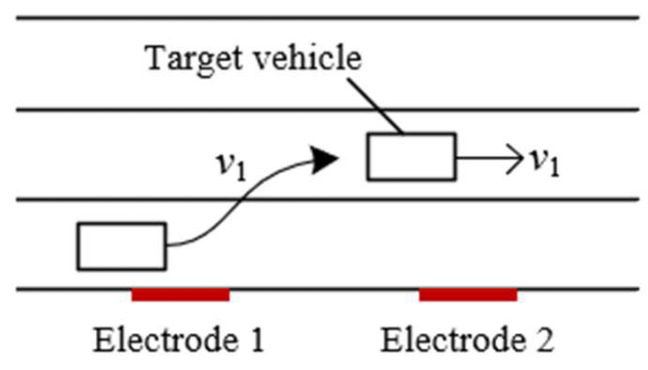
Diagram of lane changing.

**Figure 13:**
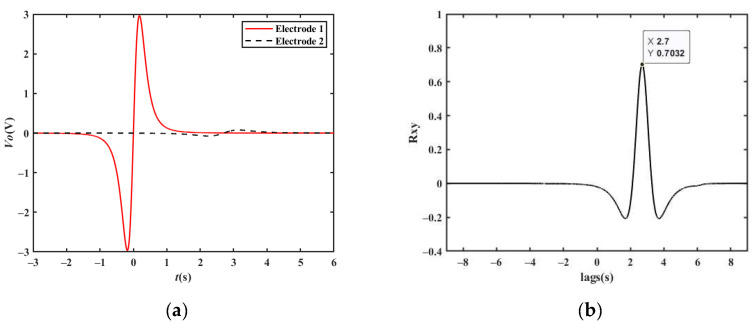
Simulation results of lane changing of a single vehicle: (**a**) simulation results at a velocity of 20 km/h; (**b**) cross-correlation results at a velocity of 20 km/h.

As shown in Figure 12, when a vehicle changes lanes between the two electrodes at a constant velocity, the corresponding output waveform is the same shape as that of a vehicle driving straight on, except for the amplitude, which means that the proposed method is effective for this situation. Figure 13 shows a simulation waveform of a car with a velocity of 20 km/h changing from lane 1 to lane 2, where the magnetic moment *m_m_* of the car is set to 1 A/m^2^, the velocity *v* = 20 km/h, the electrode area *A* = 0.01 m^2^, the capacitance *C_i_* = 10 pF, the filter circuit gain *H* = 1, the position of the detection electrode 1 is *x_1_’*=0, the position of the detection electrode 2 is *x_2_’* = 15 m, the distance between the electrode and driving lane 1 is *z_1_’* = 2 m, the distance between the electrode and driving lane 2 is *z_2_’* = 5 m, and the height of the electrode relative to the magnetic moment center *y′* = 0.1 m. According to the cross-correlation algorithm, *t_d_* is about 2.700 s, and the calculated velocity of 20.00 km/h is consistent with the set value.

#### 3.2.2. One Vehicles Followed by Another One

**Figure 14:**
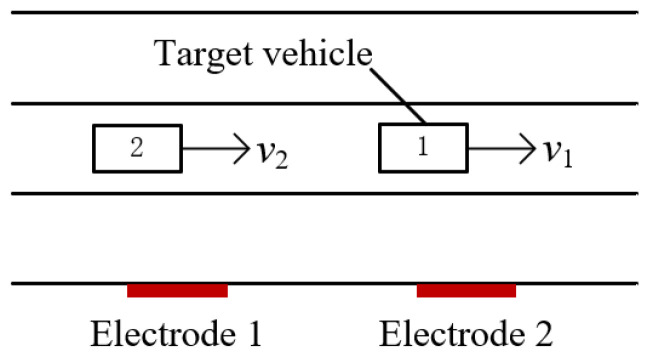
Diagram of vehicles driving close together.

In Figure 14, when two vehicles are driving close together in the same lane, one detection electrode measures two sets of waveforms for vehicle 1 and vehicle 2. When the waveforms overlap significantly, the cross-correlation technique can not evaluate the velocity correctly. Only when the detection waveforms of the two vehicles can be distinguished completely, for example, with an aliasing section of less than 50% of the waveform length, can the cross-correlation algorithm be used to derive the velocity.

**Figure 15:**
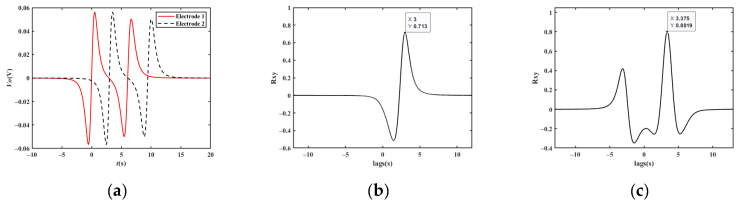
Simulation results of vehicles driving close together: (**a**) simulation results; (**b**) cross-correlation results of vehicle 1; (**c**) cross-correlation results of vehicle 2.

Assuming that the magnetic moment of the two vehicles is 1 A/m^2^, the electrode area *A* = 0.01 m^2^, the detection position of electrode 1 is *x1’* = 0, the detection position of electrode 2 is *x2’* = 15 m, the vertical distances between the electrodes and the driving lane are both *z′* = 5.25 m, the height of the electrode relative to the center of the magnetic moment *y′* = 0.1 m, the capacitance *C_i_* = 10 pF, and the filter circuit gain *H* = 1; the output waveforms of the two electrodes are given in Figure 15a for two cars driving at a distance of 27 m, with the velocity of vehicle 1 being 18 km/h and the velocity of vehicle 2 being 16 km/h, and Figure 15b,c illustrate the cross-correlation results, respectively. According to the figures, the *t_d_* values are 3.000 s and 3.375 s, and the computed velocity is consistent with the value given above.

#### 3.2.3. Multi-Vehicle Situation

**Figure 16:**
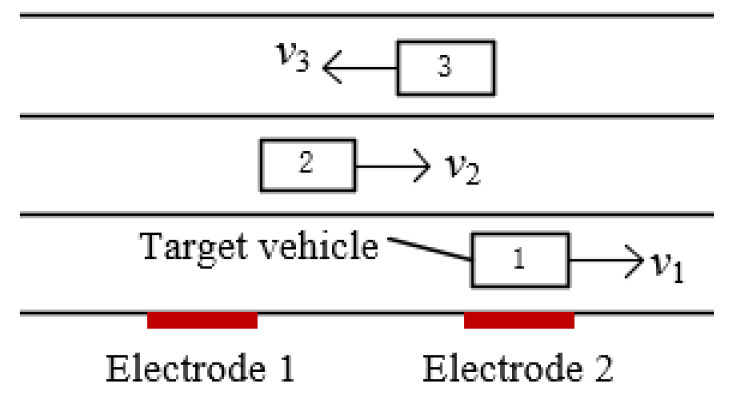
Diagram of multiple vehicles moving in multiple lanes.

Figure 16 shows a situation where the target vehicle is traveling in one lane and multiple vehicles are traveling in the other lanes at the same time.

If only one group of two electrodes is used to measure velocity, then the detection waveform is superimposed when no more than one vehicle body passes by them, resulting in the inability to assess velocity.

**Figure 17:**
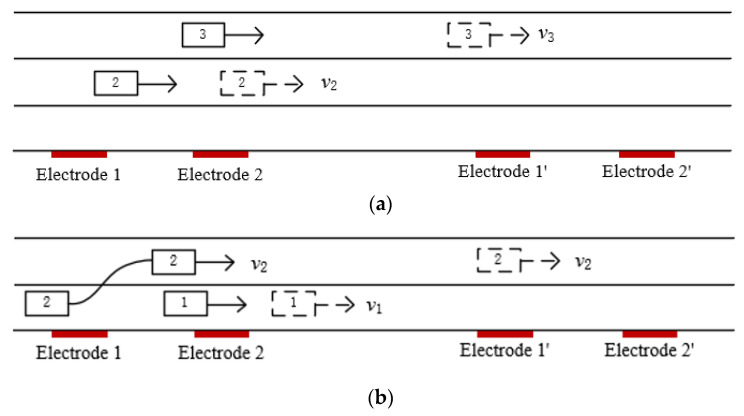
Diagram of two groups of electrodes for two scenarios: (**a**) two vehicles driving in different lanes; (**b**) changing lanes and overtaking.

In this case, another group of two electrodes must be added, as indicated in Figure 17a. Because of the difference in vehicle velocity, assuming that the velocity of vehicle 3 is larger than that of vehicle 2, after traveling for a particular amount of time, vehicle 3 will certainly overtake vehicle 2; hence, vehicle 3 will win. The waveforms of vehicles 3 and 2 can be measured using the two sets of electrodes. This method of using two groups of electrodes is equally relevant to the overtaking situation depicted in Figure 17b. Even if the waveforms of the two vehicles overlap due to them passing by the first group of electrodes in quick succession, the velocity difference between the two vehicles will make the second set of waveforms obtained by the electrodes distinguishable, allowing the velocity to be measured correctly.

**Figure 18:**
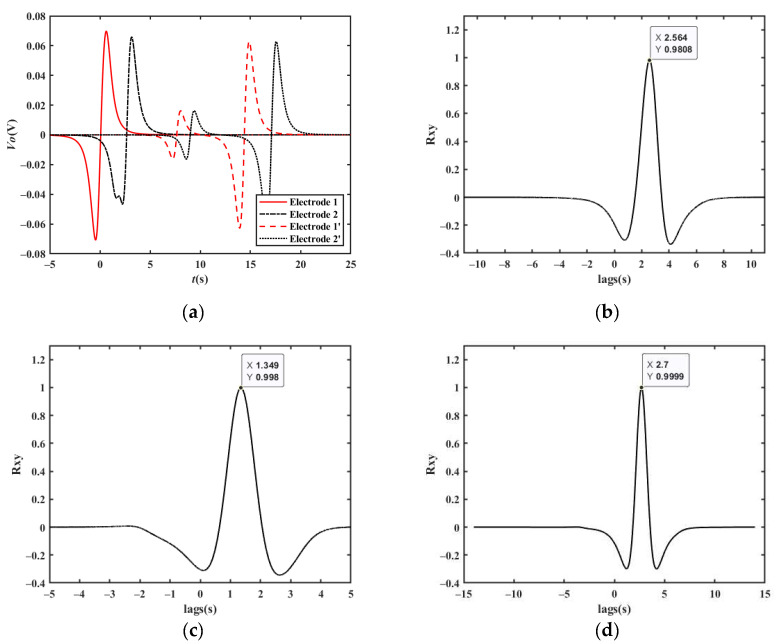
Simulation results of two vehicles driving in two lanes: (**a**) simulation results; (**b**) cross-correlation results of the first group of electrodes; (**c**) cross-correlation results of the second group of electrodes (vehicle 2); (**d**) cross-correlation results of the second group of electrodes (vehicle 3).

Figure 18 illustrates the waveform simulation diagram in the case of Figure 17a, where vehicle 3 is 5 m behind vehicle 2; vehicle 2 is traveling at a velocity of 20 km/h in lane 2; vehicle 3 is traveling at a velocity of 40 km/h in lane 3; the magnetic moment of the two cars is 1 A/m^2^; the electrode area *A* = 0.01 m^2^; the distance between the electrodes in the same group is 15 m; the distance between the two detection electrode groups is 65 m; the vertical distances z′ between the electrodes in the lane of vehicle 2 and the lane of vehicle 3 lane are 5.25 m 8.75 m, respectively; the height of the electrode relative to the center of the magnetic moment of the vehicle body *y′* = 0.1 m; the capacitance *C_i_* = 10 pF; and the filter circuit gain *H* = 1. The waveforms of the first group of electrodes are aliased, and the cross-correlation results shown in Figure 18b lead to large errors in velocity. Fortunately, the waveforms of the second set of electrodes are clear, allowing the velocities of the two cars to be measured effectively, as shown in Figure 18c,d. The overtaking simulation waveforms are similar and are not replicated.

In summary, the method proposed in this paper can achieve the velocity measurements of a single vehicle changing lanes and multiple vehicles driving in multiple lanes at the same time by using two groups of electrodes to avoid the overlapping of the velocity measurement waveforms of different vehicles. However, if the waveforms overlap as the vehicles pass by the two groups of electrodes, it is impossible to obtain the correct velocity. Furthermore, the velocity measurement method given in this paper is unaffected by light, rain, and fog, and it is immune to occlusion, which is verified in experiments.

## 4. Experimental Results

### 4.1. Experimental Setup

**Figure 19:**
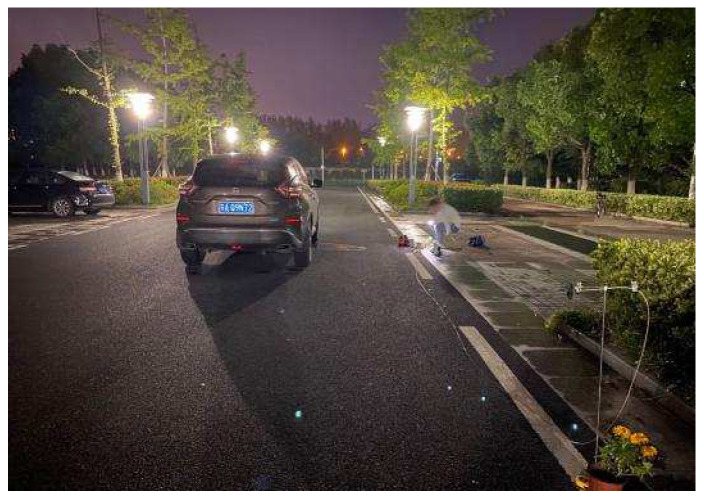
Velocity measurement experiment environment.

One group of electrodes is placed alongside the road, with their surfaces parallel to it, at a height of 0.75 m and a spacing of 15 m. The detection frontend is connected to the two electrodes, and the circuit output is connected to an oscilloscope. The velocity measurement results are acquired by processing the data through cross-correlation processing. Figure 19 shows the experimental scene, where one vehicle can be seen driving past the electrodes placed alongside the road.

### 4.2. Results

In order to further verify the feasibility of the velocity measurement method, experiments are carried out in sunny weather, windy and rainy weather, and a night environment. In the experiment, the actual vehicle velocity is obtained from the velocity value given by the vehicle. According to general knowledge, the displayed velocity of the vehicle is slightly higher than the actual driving velocity.

#### 4.2.1. Sunny Weather

**Figure 20:**
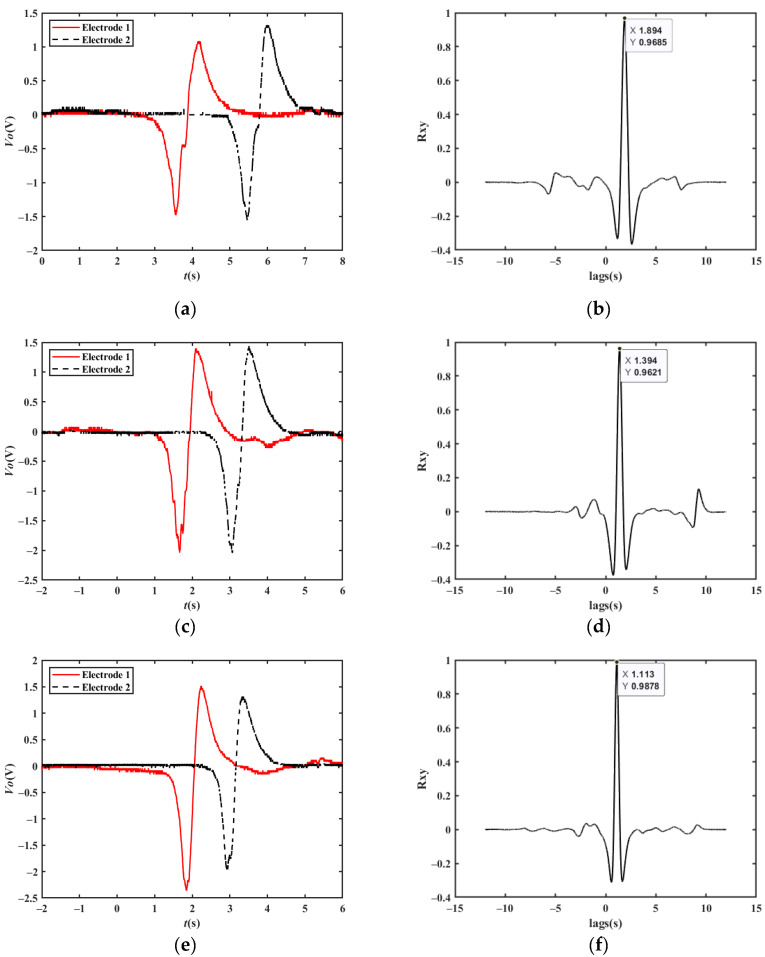
Experimental results at different vehicle velocities: (**a**) original waveforms at a velocity of 30 km/h; (**b**) cross-correlation results at a velocity of 30 km/h; (**c**) original waveforms at a velocity of 40 km/h; (**d**) cross-correlation results at a velocity of 40 km/h; (**e**) original waveforms at a velocity of 50 km/h; (**f**) cross-correlation results at a velocity of 50 km/h.

Figure 20 shows the measured velocity waveforms of a car driving at velocities of 30 km/h, 40 km/h, and 50 km/h under normal conditions; the cross-correlation results are 28.51 km/h, 38.74 km/h, and 48.52 km/h, respectively.

**Figure 21:**
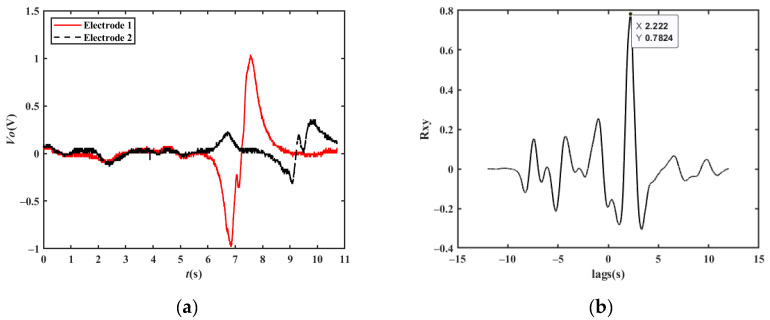
Experimental results of a single vehicle changing lanes at 25 km/h: (**a**) original waveforms at a velocity of 25 km/h while changing lanes; (**b**) cross-correlation results at a velocity of 25 km/h while changing lanes.

Figure 21 shows the acquired waveform of a single vehicle changing lanes at a velocity of 25 km/h, and the cross-correlation result is 24.30 km/h.

#### 4.2.2. Windy and Rainy Weather

**Figure 22:**
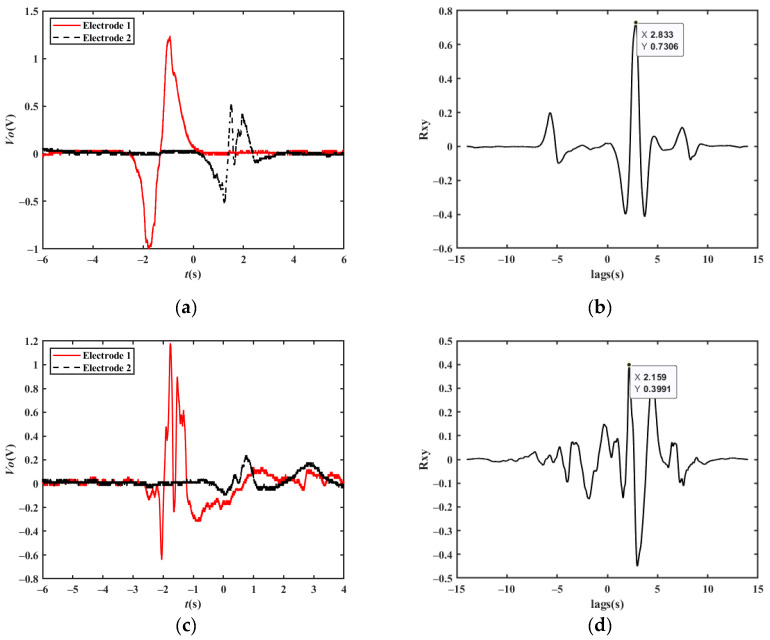
Experimental results in windy and rainy weather: (**a**) original waveforms at a velocity of 20 km/h; (**b**) cross-correlation results at a velocity of 20 km/h; (**c**) original waveforms at a velocity of 26 km/h with changing lanes; (**d**) cross-correlation results at a velocity of 26 km/h with changing lanes.

Figure 22a shows a measured waveform of a vehicle traveling at 20 km/h, with a cross-correlated velocity of 19.06 km/h; Figure 22b shows a measured waveform of a vehicle changing lanes at 26 km/h. The maximum velocity is 25.01 km/h. The results show that rain and wind may cause some waveform jitter, but they have little effect on the velocity measurement results. A waterproof cover can be attached to the electrodes to further reduce environmental disturbances.

#### 4.2.3. Night Environment

**Figure 23:**
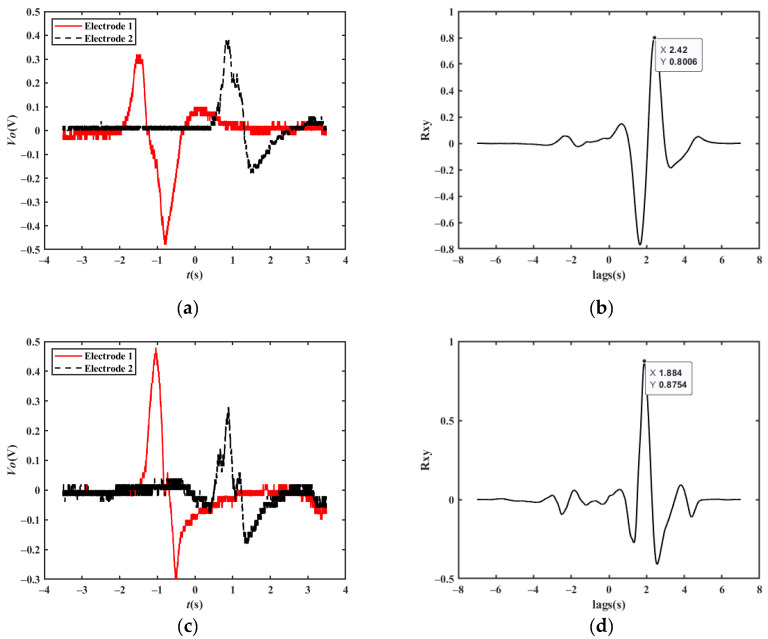
Experimental results in night environment: (**a**) original waveforms at a velocity of 23 km/h; (**b**) cross-correlation results at a velocity of 23 km/h; (**c**) original waveforms at a velocity of 30 km/h with changing lanes; (**d**) cross-correlation results at a velocity of 30 km/h with changing lanes.

Figure 23a shows the experimental results and the cross-correlated results of a vehicle moving at 23 km/h, and the cross-correlation result is 22.31 km/h. Figure 23b shows the experimental results and the cross-correlated results of a vehicle changing lanes at 30 km/h, and the cross-correlation result is 28.66 km/h. According to the actual measurement data, the night environment has no effect on the velocity measurement results.

## 5. Conclusions

The technology developed to measure velocity has become mature, and there are various types that can be used, including active radar technology, laser technology, passive ground-sensing technology, and image technology. It is difficult to satisfy multiple factors at the same time with the current velocity measurement technologies, such as accuracy, equipment cost, algorithm complexity, and anti-interference ability. Therefore, this paper proposes a passive method for measuring velocity by inducing charge changes based on the principle of the charge induction of a single vehicle changing lanes and multiple vehicles driving in multiple lanes at the same time. Compared with video technology, radar technology, and ultrasonic technology, the proposed method adopts a cross-correlation algorithm to obtain the time characteristics in order to achieve the velocity measurement; the algorithm is simple and easy to implement at a low cost. Moreover, the proposed method is completely passive, unaffected by light and rain, and immune to occlusion. The significant advantages of this method are that it can work effectively in the passive mode whether it is day or night, it is less affected by light and weather, and it can measure the velocity of vehicles changing lanes; it also has a low cost and is easy to implement.

Section 1 introduces the technical background of the velocity measurement field. In Section 2, the waveform of the induced charge change on the electrode surface caused by a vehicle is theoretically deduced and verified by experiments. In Section 3, the velocity measurement method is given, and the simulation results of several complex situations are analyzed. In Section 4, the experimental results are implemented to prove that the method is feasible.

According to the experimental results, the aim of future work should be to carry out experiments for multiple vehicles and to design a signal processing circuit in order to obtain velocity measurements in real time.

## Data Availability

Not applicable.
